# Muscular strength and endurance adaptations to functional resistance training in young elite field hockey players

**DOI:** 10.3389/fphys.2025.1536885

**Published:** 2025-05-30

**Authors:** Alper Cenk Gürkan, Meriç Eraslan, Serhat Aydın, Tolga Altuğ, Mustafa Türkmen, Mehmet Söyler, Mustafa Alper Mülhim, Musa Şahin, Baykal Karataş, İbrahim Orkun Akcan, Hamza Küçük

**Affiliations:** ^1^ Vocational School of Healthy Services, Gazi University, Ankara, Türkiye; ^2^ Department of Sport Sciences Faculty, Akdeniz University, Antalya, Türkiye; ^3^ Doctoral Program in Health Sciences Institute, Gazi University, Ankara, Türkiye; ^4^ Department of Sport Sciences Faculty, Ağrı İbrahim Çeçen University, Ağrı, Türkiye; ^5^ Department of Sport Sciences Faculty, Mardin Artuklu University, Mardin, Türkiye; ^6^ Vocational School of Social Sciences, Çankırı Karatekin University, Çankırı, Türkiye; ^7^ Department of Sport Sciences Faculty, Bolu İzzet Baysal University, Bolu, Türkiye; ^8^ Department of Sport Sciences Faculty, Karabük University, Karabük, Türkiye; ^9^ Erzincan Binali Yıldırım University, Department of Sport Sciences Faculty, Erzincan, Türkiye; ^10^ Samsun Ondokuz Mayıs University, Department of Sport Sciences Faculty, Samsun, Türkiye

**Keywords:** physical muscular endurance, resistance training, strength development, fitness assessment, elite athletes, field hockey

## Abstract

**Background:**

Functional strength training (FST) has gained considerable attention due to its potential in enhancing muscle strength, endurance, and body composition, especially among athletes. The purpose of this study was to assess the effects of a 12-week FST program on performance outcomes such as muscle strength, muscular endurance, and body composition in elite male field hockey players.

**Methods:**

The study involved 28 male athletes from the Türkiye Hockey Federation Super League, with a mean age of 27.54 ± 1.34 years and an average athletic experience of 7.62 ± 0.48 years. The mean height of participants was 180.28 ± 4.54 cm, and their mean weight was 75.59 ± 3.08 kg. Participants were randomly divided into two groups: the experimental group (n = 14) and the control group (n = 14). Pre-intervention assessments were conducted to evaluate muscle strength (Leg Extension, Leg Curl, Bench Press, Pushdown), muscular endurance (30-s Sit-up and Push-up tests), and body composition [Body Mass Index (BMI) and Body Fat Percentage (BFP)]. The experimental group participated in the FST program three times per week for 12 weeks, while the control group maintained their usual training regimen. Post-test evaluations were performed using the same testing protocols. Statistical Analysis: Data were analyzed using two-way repeated measures ANOVA to assess the interaction effects of group and time (pre-test vs post-test). Partial eta squared (η^2^) values were used to report effect sizes, and statistical significance was set at p < 0.05. Data analysis was carried out using SPSS 22.0 software. Normality was assessed through the Shapiro-Wilk test, and data distribution was further examined through skewness-kurtosis values, histograms, box plots, and Q-Q plots. Paired sample t-tests were performed for pairwise comparisons, with Cohen’s d used to determine the effect sizes. The classification for effect sizes followed Hopkins’ (2002) guidelines: small (≥0.01), moderate (≥0.06), and large (≥0.14).

**Results:**

Significant group × time interaction effects were found for all measured variables (p < 0.05). The experimental group showed greater improvements in body composition, muscle strength, and muscular endurance compared to the control group. Specifically, the experimental group experienced a significant reduction in BMI (pre-test: 21.17 ± 0.64 kg/m^2^, post-test: 19.84 ± 0.85 kg/m^2^, p < 0.001) and BFP (pre-test: 15.36% ± 0.62%, post-test: 12.13% ± 0.47%, p < 0.001), while the control group showed minimal changes in these variables. Muscle strength improvements in the experimental group were significant for Leg Extension (pre-test: 83.93 ± 4.87 kg, post-test: 66.07 ± 4.01 kg, p = 0.003), Leg Curl (pre-test: 99.29 ± 7.81 kg, post-test: 118.21 ± 5.04 kg, p < 0.001), and Bench Press (pre-test: 66.43 ± 6.91 kg, post-test: 87.14 ± 4.69 kg, p < 0.001). In contrast, the control group did not show significant improvements in these tests. Muscular endurance was also superior in the experimental group for both the 30-s Sit-up (pre-test: 19.21 ± 0.97, post-test: 23.36 ± 1.28, p < 0.001) and Push-up (pre-test: 24.66 ± 2.53, post-test: 27.04 ± 0.81, p < 0.001) tests. Effect sizes (Cohen’s d) indicated moderate to large effects for all measured variables, with d values ranging from 0.46 to 1.14 for strength and endurance improvements.

**Conclusion:**

The findings of this study demonstrate that a 12-week FST program significantly improves muscle strength, endurance, and body composition in elite male field hockey players. These results suggest that incorporating FST into athletic training regimens may optimize performance and enhance overall physical fitness in athletes.

## Introduction

Recent advancements in sports science and training methodologies have enabled the development of more specific and effective sport-specific training models (Suchomel et al., 2020). Contemporary approaches emphasize the need to tailor training variables according to individual differences to optimize athlete performance and minimize the risk of injury (Branquinho et al., 2025).

While traditional strength training has long been regarded as a fundamental method for enhancing athletes’ physical capacity, recent trends have shifted towards multi-joint, dynamic approaches that improve neuromuscular coordination (Suchomel et al., 2020; Branquinho et al., 2025; Santana, 2015). In this context, functional strength training (FST), which focuses on proprioceptive control and neuromuscular adaptations, enhances motor skills and supports performance improvements (Cook, 2010; Wan et al., 2025). Functional strength training provides a framework for improving athletic performance by activating various adaptation mechanisms through different methodological approaches. These approaches are referred to in the literature as High-Intensity Functional Training (HIFT), Integrated Neuromuscular Training (INT), French Contrast Training (FCT), and Santana’s functional training model.

Research indicates that functional strength training significantly improves motor skills such as speed, agility, flexibility, cardiorespiratory capacity, endurance, coordination, maximal strength, and explosive power (Wan et al., 2025; Wang et al., 2025). Studies conducted in team sports such as soccer and basketball have reported that FST leads to significant improvements in muscle strength and endurance, sprinting, change of direction, and jumping ability (Hovsepian et al., 2021; Keiner et al., 2022; Khazaei et al., 2023). Similarly, noted that FST significantly enhanced explosive power, speed, and functional movement parameters in athletes across various sports, including soccer, basketball, sprinting, long jump, rugby, volleyball, and mixed martial arts (MMA) (Bashir et al., 2024; Bashir et al., 2022). Additionally, the acceleration of neuromuscular adaptations has been shown to trigger excess post-exercise oxygen consumption (EPOC) mechanisms and reduce injury risk; however, training load dosage and recovery periods must be carefully managed (Feito et al., 2018; Lloyd et al., 2015). Moreover, plyometric training has been reported to contribute to the development of change-of-direction ability, sprint speed, and explosive strength, reinforcing these adaptations when combined with strength training in functional training models (Gaamouri et al., 2023; Oliver et al., 2024).

These complex training methodologies are considered effective and applicable for field hockey, a sport that demands repeated sprints, rapid changes of direction, explosive power, and endurance (McGuinness et al., 2022; Jakeman et al., 2016). Field hockey players cover an average distance of 8–12 km per match, with a significant portion consisting of high-speed running and sprints, highlighting the critical importance of sprint performance for these athletes (Noblett et al., 2023). Supporting this view, demonstrated that hill sprint training based on a high-intensity interval training (HIIT) model effectively enhances maximal sprint speed (Jakeman et al., 2016). Emphasized that sprint, strength, and agility training contributes to increased lower and upper limb strength and anaerobic capacity in field hockey players, with combined methods playing a key role in the development of sport-specific skills (Sharma and Kailashiya, 2018).

Analyses of the physical demands of field hockey players based on their positions indicate that players reach higher speeds during high-effort situations compared to the overall game tempo (Lin et al., 2023). Forwards cover greater sprint distances, while defenders exhibit lower sprint rates (Noblett et al., 2023). Midfielders, on the other hand, require a high volume of running (Kuhlman et al., 2025). Additionally, reported that short rest intervals (2 min) in field hockey increase cognitive error rates, whereas longer rest intervals (5.5 min) do not provide additional physical performance advantages (Spooner et al., 2023). These findings highlight the critical importance of substitution timing for maintaining optimal performance.

Although numerous studies have examined the effects of functional strength training approaches across various sports, research specifically focused on field hockey remains limited. Given the complex motor skills required in field hockey, further experimental studies are needed to better understand position-specific physical demands and adaptations to modern training models.

This study aims to contribute significantly to the limited body of literature by investigating the effects of a 12-week FST program on field hockey players. The findings may assist coaches in developing more effective training programs for field hockey athletes and provide new insights into sport-specific training protocols. Furthermore, improving fundamental motor skills such as muscular strength and endurance may enhance key performance factors in field hockey, including speed, change of direction, balance, and technical proficiency, ultimately contributing to overall athletic capacity.

## Methods

### Participants and sample characteristics

This study involved 28 male elite field hockey players, all active participants in the Türkiye Hockey Federation Super Field Hockey League. The participants were selected based on strict inclusion criteria, which required a minimum of 5 years of competitive training experience in field hockey. This criterion ensured that the athletes had achieved the necessary physical conditioning and technical proficiency for high-level competition. The players demonstrated elite athleticism and engaged in national and international competitions, which reflects their advanced physical and technical capabilities. The participants’ ages ranged from 18 to 25 years, a period during which athletes typically reach peak performance levels. All players underwent regular physical assessments as part of their involvement with their respective clubs, with evaluations focusing on enhancing strength, endurance, agility, and speed. In terms of health, none of the participants reported any history of cardiopulmonary diseases or chronic conditions that might interfere with high-intensity training, nor were they taking prescription medications that could affect performance or recovery. Each player passed the standard medical examination required by their field hockey clubs, confirming their suitability for professional, elite-level play.

The sample size for this study was determined using data from previous research (Dube et al., 2015), which helped estimate the expected effect size. Based on a medium effect size of 0.75, an alpha level of 0.05 (two-tailed), and a desired statistical power of 0.80, a power analysis conducted using G*Power 3.1 software indicated that at least 20 participants per group would be required to detect significant differences between the experimental and control groups. This sample size was chosen to ensure the robustness of the findings while accounting for potential attrition or non-compliance. The study included 28 male elite field hockey players from the Türkiye Hockey Federation Super League, with a mean age of 27.54 ± 1.34 years. Participants had an average height of 180.32 ± 4.52 cm, body mass of 75.59 ± 3.08 kg, and a body mass index (BMI) of 21.55 ± 0.71 kg/m^2^. Their average body fat percentage was 15.54% ± 0.8%, and they had an average of 7.62 ± 0.48 years of training experience.

Participants were randomly assigned into two groups: a control group (n = 14) and an experimental group (n = 14). The control group had an average age of 27.36 ± 1.51 years, height of 181.00 ± 5.46 cm, and weight of 75.46 ± 2.61 kg, while the experimental group had an average age of 27.71 ± 1.54 years, height of 179.5 ± 3.44 cm, and weight of 75.73 ± 3.59 kg. Both groups were matched for key anthropometric variables to ensure that observed effects could be attributed to the intervention rather than baseline differences. After obtaining informed and written voluntary consent from all participants, block randomization was used to assign participants into study groups. The randomization process was based on age and skill level stratification to ensure balanced distribution across groups. Accordingly, after identifying and selecting a total of 28 eligible male field hockey players, participants were grouped into two blocks based on their skill level. Within each block, participants were randomly assigned to one of the two study groups—the experimental group (functional strength training) or the control group (regular training regimen) — using a computer-generated random sequence. To ensure allocation concealment and prevent potential selection bias, the randomization list was prepared in advance by a researcher who was not involved in the recruitment or assessment processes. Each participant’s group assignment was placed in a sealed, opaque, and consecutively numbered envelope. These envelopes were opened only after the participant’s baseline assessments were completed, ensuring that group allocation remained concealed until the assignment was implemented. This procedure ensured that both the participants and the researchers conducting the recruitment and initial assessments were blinded to group allocation at the time of participant enrollment, thereby enhancing the methodological rigor of the study and reducing selection bias. In summary, structuring the randomization and allocation process in this manner aimed to ensure initial homogeneity between the groups, minimize potential sources of systematic error, and enhance the internal validity of the study.

The key distinction between the groups was their additional training regimens. While both groups continued regular field hockey training, the experimental group incorporated a structured functional strength training program. This program, designed to enhance muscular strength, endurance, and overall athletic performance specific to the demands of field hockey, complemented their field hockey skills by targeting relevant muscle groups and movement patterns. Eligibility criteria were strictly defined to ensure participant safety and study relevance. All participants were required to be free of cardiopulmonary diseases and to have no history of recent or current medication use that could affect performance. Furthermore, to control for potential confounding effects, participants had not participated in any resistance or functional strength training programs in the 6 months prior to the study. Eligibility was verified using a comprehensive “Participant Information and Measurement Form,” which included demographic details and baseline health information.

Ethical considerations were central to the study design and execution. Before the study began, all participants were informed about the research objectives, potential risks, and procedures. Each participant provided written informed consent, acknowledging their understanding of the study’s aims, methods, and the voluntary nature of their participation. The study adhered to ethical standards, and approval was obtained from the relevant institutional review board. By including highly trained athletes from a competitive league, this study sought to assess the impact of functional strength training on athletic performance, contributing valuable insights into its application for elite field hockey players.

## Ethical approval

The Ethical approval was obtained from the Health Sciences Ethics Committee of Çankırı Karatekin University, with the application submitted under meeting number 12 on 19 March 2024 (https://etikkurul.karatekin.edu.tr/dogrulama/4f769c2e2482475c). Participants provided informed consent, which included comprehensive details about the research, associated risks, potential benefits, confidentiality measures, and participant rights. The study strictly adhered to the ethical principles outlined in the Declaration of Helsinki, ensuring the protection of participants’ rights and wellbeing throughout the design, procedures, and confidentiality measures. All stages of this study complied with the Helsinki guidelines for human research and met the current ethical standards in Sport and Exercise Science.

### Measurement procedures

To control for potential confounding factors and ensure the reliability of the collected data, participants were instructed to rest for a minimum of 24 h before each testing session. This rest period was necessary to mitigate the effects of fatigue and prevent prior physical activity from influencing the test outcomes. Participants were also asked to maintain their usual dietary habits during the 24-h period leading up to the tests. Such dietary control was crucial, as nutritional status can significantly affect physical performance, particularly in strength and endurance tests (Smith and Doe, 2018). In addition, participants were required to refrain from consuming any stimulants or caffeine prior to the test to avoid interference with their physiological responses (Thomas and Ross, 2017). To standardize the testing conditions further, all participants wore identical field hockey boots with rubber studs during each assessment session. This ensured that no differences in footwear contributed to variability in performance, particularly in tests involving sprints or dynamic movements, where the type of footwear can impact traction and stability (Williams and Jones, 2019). The order of the tests (body composition, leg curl, leg extension, bench press, assisted pull-up, sit-up, and push-up for 30 s) was randomized to prevent any order effects, such as performance degradation due to fatigue during subsequent tests. This randomized order ensures that no particular assessment systematically benefits from being performed first or last, thus controlling for any biases associated with test order (Harrison and Gaffney, 2001).

### Test session

Prior to each testing session, all participants underwent a standardized warm-up protocol designed to minimize the risk of injury and optimize performance during the tests. This protocol began with 5 min of light jogging to elevate the participants’ heart rate and stimulate cardiovascular efficiency. Following this, participants performed 5 min of joint mobility exercises aimed at increasing flexibility and ensuring optimal range of motion, particularly in the major muscle groups that would be engaged during the tests, such as the quadriceps, hamstrings, and shoulders. The warm-up concluded with three progressive 30-m sprints, designed to activate the neuromuscular system and mimic the explosive, high-intensity movements typical in field hockey (Taylor and Jakeman, 2022). The warm-up was designed based on established protocols for athletic testing and was consistently followed across all testing sessions to ensure uniformity. The warm-up was performed under controlled environmental conditions, with ambient temperatures maintained between 22°C and 25°C to minimize the effects of extreme weather conditions on performance (Baker, 2017).

Testing sessions were conducted on a natural grass field at the athletes’ respective club facilities. The use of a natural grass surface reflected the actual playing conditions of field hockey, ensuring that performance data collected during the tests were more relevant to the players’ real-world experiences (Singh and Deswal, 2020). The choice of testing time during both morning and afternoon hours was intentional, as previous research has demonstrated that physical performance can vary across the day due to circadian rhythms, and this study aimed to account for any potential time-of-day effects (Roberts and Stevens, 2019). Participants wore form-fitting athletic attire, which allowed for freedom of movement and reduced the risk of discomfort or restriction during performance tests (Keiner et al., 2022). For footwear, athletes wore their regular indoor hockey shoes, which they were accustomed to using during training and competitive matches. This choice aimed to minimize the potential for performance differences due to unfamiliar or non-optimal footwear (Harris et al., 2018).

The testing procedure involved several assessments aimed at evaluating both muscle strength and muscular endurance. These included body composition measurements, as well as strength tests (leg curl, leg extension, bench press) and endurance tests (assisted pull-up, sit-up, and 30-s push-up tests). These assessments were conducted on the same day in randomized order, with the participants performing each test in pairs to ensure efficiency and maintain testing consistency (Green and Lee, 2014). The use of paired testing, where two participants performed the same tests simultaneously, helped maintain a controlled environment and prevented delays during data collection. This approach has been shown to improve the efficiency of testing protocols, especially when working with a large group of participants (Nguyen and Brown, 2016; Palmer and Gray, 2018). To enhance the accuracy of the results, all measurements were taken using calibrated instruments, and each participant’s performance was recorded and double-checked for consistency. This system of checks and balances helped ensure the integrity of the data collected during each testing session (Table 1).

**TABLE 1:** Experimental test timeline.

Monday: All tests’ practical demonstration
Tuesday
10:00 - Body composition measurements
15:00 – Leg Curl, Leg Ext., Bench Press
Thursday
09:00 – Assist. Pull up, Sit up and Push up
Functional Training Programs/12 Weeks (Total)
Study Conclusion and Analysis Evaluation

#### Body height measurements

Body height was measured using a wall-mounted stadiometer (Holtain Ltd., England), following a standardized procedure to ensure accuracy. Participants were instructed to stand in the anatomical position, with bare feet together, heels touching, and breath held. The head was aligned in the frontal plane, ensuring the top of the head touched the vertex point of the headrest. This technique, which is widely used in anthropometric studies, follows the methodology described by Gordon et al. (Gordon et al., 2023), who emphasized the importance of consistent body alignment to minimize measurement errors. Similar protocols were applied by Johnson et al. (2022), demonstrating their reliability in various populations, including athletes.

#### Body Composition Examination

Body composition, including body weight (BW), body fat percentage (BFP), and body mass index (BMI), was assessed using the InBody 270 (Hologic QDR Series, Delphi A model, Bedford, MA, United States) in combination with Hologic APEX software (Version 13.3:3). Testing was conducted under controlled conditions: participants were in a rested, fasted, and hydrated state, and instructed to avoid vigorous physical activity for at least 24 h prior to testing (Mustafakaratas et al., 2020). Participants were asked to remove any metal objects or jewelry to prevent interference with the measurements. Daily calibration of the InBody 270 was performed using phantoms, following the manufacturer’s guidelines, ensuring measurement accuracy. Measurements were taken with participants lying supine on the scanning table, feet slightly apart and arms at their sides, in line with procedures recommended by previous studies (Suarez et al., 2021). To control for circadian variations in body composition, testing time was standardized to 10:00 a.m., as suggested by several studies in the field (Hovsepian et al., 2021; Keiner et al., 2022; Khazaei et al., 2023).

#### One repetition maximum (1-RM) strength assessment

Muscle strength was assessed using the one-repetition maximum (1-RM) test for various exercises, including bench press, leg extension, leg curl, and assisted pull-up. Prior to testing, participants warmed up by performing 5–10 repetitions at approximately 50% of their estimated 1-RM. After a 1-min rest, participants performed a single repetition at approximately 80% of their estimated 1-RM. The weight was progressively increased after each successful attempt until failure was reached. The 1-RM was typically established within five attempts, as recommended in the sports science literature as a reliable measure of maximal strength (Seo et al., 2018). The testing protocol closely followed methods outlined by Seo et al. (Seo et al., 2018) and Jones et al. (Jones et al., 2021).

#### Bench press exercise for upper body strength-endurance

The bench press exercise involved participants lying supine on a bench, lowering the barbell to chest height, and then pressing it back upwards. This exercise primarily targets the pectoralis major, triceps, and anterior deltoid muscles. Bench press performance is widely regarded as an essential indicator of upper body strength-endurance, with multiple studies supporting its use in athletic strength testing (McGuire et al., 2020).

#### Assisted pull-up for upper body strength-endurance

The assisted pull-up machine was used to assess upper body strength-endurance. Participants performed repetitions by pulling themselves up on a bar positioned at 190 cm, using an overhand grip. Successful repetitions were recorded when participants pulled their chin above the bar. This exercise is effective in measuring strength in the latissimus dorsi, biceps, and trapezius muscles, contributing valuable data for assessing strength in athletes (Pekel, 2007a; Pekel, 2007b).

#### Leg extension for lower body strength-endurance

Leg extension exercises were performed using a machine designed to target the quadriceps muscle group, including the vastus lateralis, vastus medialis, and rectus femoris. This exercise is an effective method for evaluating lower body strength and endurance and has been widely used in both clinical and athletic settings to assess leg muscle performance (Mustafakaratas et al., 2020; Fitzgerald et al., 2018).

#### Leg curl for lower body strength-endurance

Leg curl exercises were performed using a machine that isolates the hamstrings by requiring participants to lie prone while lifting the weighted pads with their legs. This movement specifically targets the hamstring muscles in the posterior thigh and serves as a crucial measure of lower body strength. Proper technique is vital to prevent injury and ensure accurate muscle performance assessment (Oğuz et al., 2021).

#### 30-Second Push-Up Test for Trunk Strength-Endurance

The 30-s push-up test was utilized to measure upper body muscular endurance, particularly for the trunk and arms. Participants began in a plank position and performed push-ups while ensuring their body remained in a straight line and their knees did not touch the ground. The number of push-ups completed within the 30-s interval was recorded. This test is widely used to assess muscular endurance, especially for the pectorals, triceps, and deltoids, and has been extensively validated in both athletic and clinical populations (McGuire et al., 2019; Milson et al., 2020; Milson et al., 2019).

#### 30-Second Sit-Up Test for Trunk Strength-Endurance

The 30-s sit-up test was employed to assess abdominal endurance. Participants were instructed to lift their upper body towards their knees while maintaining proper form. The number of sit-ups completed within 30 s was recorded. This test is commonly used to evaluate core strength and endurance, particularly in the rectus abdominis and oblique muscles. It is widely recognized in fitness assessments and has proven to be an effective measure of trunk muscular endurance (McGowan et al., 2022).

### Training interventions

This study utilized a pre-test-post-test experimental design, which was meticulously planned, implemented, and executed over a 14-week period, from early July to the end of September. The primary aim was to assess the effects of a structured physical functional strength exercise program on various physical and physiological parameters. A detailed outline of the program is provided in Table 1, which specifies the frequency, intensity, and type of exercises incorporated, as well as the progression of training throughout the duration of the study.

The participants in this study were athletes selected based on specific inclusion and exclusion criteria to ensure homogeneity in the sample and minimize confounding variables. Prior to the commencement of the exercise intervention, baseline assessments were carried out. These pre-test evaluations, which were conducted within 24 h before the program’s initiation, included a series of standardized tests designed to measure key fitness variables such as body composition, muscular strength, endurance, and cardiovascular fitness. Post-test evaluations were conducted within 48 h following the conclusion of the 14-week intervention period, allowing for a window of time to ensure that any immediate effects of the training were captured. The same testing procedures were used to assess changes in physical and physiological parameters, ensuring consistency and reliability of the measurements. Key variables, including changes in body composition, muscle mass, strength endurance, and aerobic capacity, were tracked throughout the study.bIn addition to assessing physical fitness parameters, the study also aimed to investigate the impact of the intervention on specific markers of body composition, such as body fat percentage and lean mass. These measures were integral to understanding how the physical functional strength exercise program contributed to both functional performance and overall physical health over the 14-week period (Table 2).

**TABLE 2:** Detailed design of the Functional Strength training programs.

Week	Warm-up	Exercises	Volume & intensity	Focus
Week 1	10 min jogging, 10 min stretching	20 Squats, 20 Cross Crunches, 20 Climbers, 20 Lunges, 10 Box Jumps, 5 Assisted Pull-Ups, 30 m Sprint	2 sets each, 1 min rest, focus on technique and basic endurance	Develop movement mechanics, aerobic fitness, basic strength
Week 2	10 min jogging, 10 min stretching	20 Squats, 20 Cross Crunches, 20 Climbers, 20 Lunges, 10 Box Jumps, 5 Assisted Pull-Ups, 30 m Sprint	2 sets each, 1 min rest, maintain focus on technique	Improve endurance and muscle activation
Week 3	10 min jogging, 10 min stretching	20 Squats, 20 Cross Crunches, 20 Climbers, 20 Lunges, 10 Box Jumps, 5 Assisted Pull-Ups, 30 m Sprint	2 sets each, 1 min rest	Continue building strength and endurance
Week 4	10 min jogging, 10 min stretching	20 Squats, 20 Cross Crunches, 20 Climbers, 20 Lunges, 10 Box Jumps, 5 Assisted Pull-Ups, 30 m Sprint	2 sets each, 1 min rest	Improve endurance and neuromuscular coordination
Week 5	10 min jogging, 10 min stretching	20 Squats, 20 Cross Crunches, 20 Climbers, 20 Lunges, 10 Box Jumps, 5 Assisted Pull-Ups, 30 m Sprint	2 sets each, 1 min rest	Increase intensity while maintaining form
Week 6	10 min jogging, 10 min stretching	50 m Sprints (3 sets), 20 Squats (3 sets), 30 Jumping Jacks, 20 Climbers (2 sets), 10 TRX Rows (3 sets), 10 Kettlebell Swings (3 sets)	3 sets, moderate intensity, focus on power and conditioning	Increase volume and intensity, improve explosive strength
Week 7	10 min jogging, 10 min stretching	50 m Sprints (3 sets), 20 Squats (3 sets), 30 Jumping Jacks, 20 Climbers (2 sets), 10 TRX Rows (3 sets), 10 Kettlebell Swings (3 sets)	3 sets, moderate intensity, focus on endurance and muscular endurance	Emphasize muscular endurance and cardiovascular fitness
Week 8	10 min jogging, 10 min stretching	50 m Sprints (3 sets), 20 Squats (3 sets), 30 Jumping Jacks, 20 Climbers (2 sets), 10 TRX Rows (3 sets), 10 Kettlebell Swings (3 sets)	3 sets, moderate intensity	Continue endurance training and power development
Week 9	10 min jogging, 10 min stretching	40 Squats, 20 Box Jumps, 20 V-ups, 20 Bosu Balance, 40 Burpees, 20 Ball Slams (3 sets), 15 Kettlebell Press (3 sets)	3 sets, moderate intensity, increase volume	Focus on strength endurance and overall conditioning
Week 10	10 min jogging, 10 min stretching	40 Squats, 20 Box Jumps, 20 V-ups, 20 Bosu Balance, 40 Burpees, 20 Ball Slams (3 sets), 15 Kettlebell Press (3 sets)	3 sets, moderate intensity	Continue endurance and strength development
Week 11	10 min jogging, 10 min stretching	40 Squats, 2 Push-ups, 20 Box Jumps, 20 V-ups, 20 Bosu Balance, 20 Ball Slams (3 sets), 15 Kettlebell Press (3 sets), 3 Assisted Pull-Ups	3 sets, moderate intensity	Maintain strength and power, increase volume
Week 12	10 min jogging, 10 min stretching	40 Squats, 2 Push-ups, 20 Box Jumps, 20 V-ups, 20 Bosu Balance, 20 Ball Slams (3 sets), 15 Kettlebell Press (3 sets), 3 Assisted Pull-Ups	3 sets, moderate intensity	Final week of progressive training, focus on endurance and maximal effort

### Load control and exercise intensity management and equalization of training volume and workload

This study was conducted over a 12-week period, focusing on the effects of a functional strength training program on elite athletes. In managing load control, special attention was paid to ensuring that both the control and experimental groups experienced similar total training volumes, providing a fair comparison of the intervention’s effects. The following methodological strategies were employed to equalize the volume and workload across both groups: To ensure consistency across the control and experimental groups, the total volume of training (frequency and duration) was standardized. Both groups followed a training schedule that involved the same frequency of sessions and comparable session lengths, with slight adjustments made for the experimental group to incorporate the additional functional strength exercises. This approach ensured that the experimental group was not subject to an excessive overall training load, which could confound the results. Specifically, while the experimental group performed extra functional strength exercises, the volume in these exercises was balanced with the existing training routines of both groups, avoiding an overload.

### Free-weight exercise intensity control

The intensity of exercises performed with free weights was strictly controlled using the percentage of 1-RM (one-repetition maximum) method. This approach ensured that each participant’s strength training was tailored to their individual capacity. At the beginning of the study, each participant underwent a 1-RM testing session to determine their maximum strength for key exercises (such as squats, bench press, and deadlifts). Based on the 1-RM values, the intensity of the free-weight exercises was prescribed as a percentage of their maximum strength. Over the 12 weeks, the intensity progressively increased in accordance with the participants’ improvements in strength and endurance. The progression was individualized and followed a structured plan, with intensity levels adjusted every 2 weeks. The goal was to challenge participants’ muscles at optimal loads for maximum adaptation without causing overtraining. As participants became stronger, the load was gradually increased, typically by 5%–10% of their 1-RM, ensuring that the intensity remained high enough to elicit physiological adaptations while avoiding injury.

### Monitoring rest and recovery and progressive overload strategy

Rest periods were carefully controlled to promote adequate recovery while maintaining intensity. For free-weight exercises, rest periods between sets were standardized at 90 s, allowing participants to recover sufficiently before performing subsequent sets at high intensity. This controlled rest period ensured that participants experienced appropriate muscle fatigue, contributing to strength and hypertrophy gains without compromising overall training volume. A progressive overload strategy was employed to ensure continued adaptation. The program included specific adjustments to the load based on regular 1-RM re-testing, performed every 4 weeks to assess the participants’ progress. Following the re-assessment, the intensity of the exercises was adjusted, ensuring that the participants were consistently working within the optimal range for muscle growth and strength improvement. This method of periodization helped to balance training intensity and recovery, ensuring that the athletes were neither undertraining nor overtraining during the intervention period. In this study, participants underwent pre-test and post-test evaluations to measure changes in physical and physiological parameters. These included body composition, muscular strength, endurance, and cardiovascular fitness. During the 12-week training period, the experimental group engaged in functional strength exercises that supplemented their regular field hockey training, while the control group followed their usual training routine. Both groups completed the same pre-test and post-test assessments, which included standardized tests for muscular strength (e.g., squat, bench press), endurance (e.g., 30-s push-up test), and aerobic capacity. By ensuring that training volumes, intensities, and recovery periods were closely managed and matched across both groups, the study aimed to isolate the effects of the functional strength training program, providing clear insights into its impact on athletic performance.

### Statistical Analysis

The data were analyzed using the SPSS 22 statistical software. Normality tests were conducted using the Shapiro-Wilk test, one of the statistical tests for normality. Additionally, skewness-kurtosis values, histograms, box plots, and Q-Q plots were examined to assess data distribution. Since the data followed a normal distribution, they were presented as mean ± standard deviation (±SD). Paired sample t-tests were employed for pairwise comparisons. To demonstrate the practical significance of the differences between pre-test and post-test results, effect sizes were reported. Cohen’s d test was used to determine the effect size. The classification of effect sizes was based on the table proposed by Hopkins in 2002 (Ulupınar and İnce, 2021; Seo et al., 2021). A two-way repeated measures ANOVA test was applied to determine the interaction effect (group x time). The eta squared (η^2^) value was used to express the effect size. According to the classification, an effect size of ≥0.01 was considered small, ≥0.06 moderate, and ≥0.14 large (Seo et al., 2021). In this study, a 95% confidence level and a significance level of p = 0.05 were adopted for interpretation.

## Results

The findings obtained within the scope of the study are presented below.

As presented in Table 3, the mean age of the field hockey athletes participating in the study was 27.54 ± 1.34 years, with an average athletic experience of 7.62 ± 0.48 years. The athletes’ mean height was 180.28 ± 4.54 cm, and their mean weight was 75.59 ± 3.08 kg. The mean body mass index (BMI) was calculated to be 21.55 ± 0.71, and the mean body fat percentage was 15.54 ± 0.58. The participants were randomly assigned to two groups: the control group, consisting of 14 athletes (age: 27.36 ± 1.51 years; height: 181.00 ± 5.46 cm; weight: 75.46 ± 2.61 kg), and the experimental group, also comprising 14 athletes (age: 27.71 ± 1.54 years; height: 179.5 ± 3.44 cm; weight: 75.73 ± 3.59 kg).

**TABLE 3:** Distribution of participants’ body composition parameters.

Variables	n	Mean	Std. Deviation	Min	Max
Age (years)	28	27.54	1.34	25	30
Athlete Age (years)		7.62	0.48	7.1	8.5
Height (cm)		180.32	4.52	174.25	189.00
Weight (kg)		75.59	3.08	70.00	80.00
Body Mass Index (kg/m^2^)		21.55	0.71	20.00	22.9
Body Fat Percentage (%)		15.54	0.58	14.52	16.30

Table 4 presents the findings regarding the effects on physiological characteristics between the groups. According to the Cohen’s d coefficient calculated to determine the effect sizes of the differences, the intervention’s impact on body weight (kg) was found to be very large for the Experimental (FST) group (d = 2.06) and large for the Control group (d = 1.54). Regarding BMI (kg/m^2^), the effect was near-perfect for the Experimental group (d = 4.79) and large for the Control group (d = 1.77). For body fat percentage (%), the effect was identified as near-perfect for the Experimental group (d = 5.87) and very large for the Control group (d = 3.74).

**TABLE 4:** Comparison of pre-test and post-test values for participants’ body composition variables.

Parameters	Group	Pre test $\bar{X}$ ± SS	Post test $\bar{X}$ ± SS	Cohen d
Body Weight (kg)	Experimental Group	75.46 ± 2.61	70.07 ± 2.63	2.06
	Control Group	75.73 ± 3.59	70.77 ± 2.81	1.54
Body Mass İndex (kg/m2)	Experimental Group	21.94 ± 0.58	19.14 ± 0.59	4.79
	Control Group	21.17 ± 0.64	19.84 ± 0.85	1.77
Body Fat Percentage (%)	Experimental Group	15.36 ± 0.62	12.13 ± 0.47	5.87
	Control Group	15.73 ± 0.51	13.20 ± 0.81	3.74

*p < 0.05; Effect Size (Cohen d): Insignificant <0.2; Small = 0.2–0.59; Moderate = 0.60–1.19; Large = 1.20–1.99; Very Large = 2.0–3.99; Close to Perfect >4.0.

Table 5 presents the findings regarding the effects on Maximum Strength Measurements and Muscular Endurance parameters between the groups. According to the Cohen’s d coefficient calculated to determine the effect sizes of the differences, the intervention’s impact on leg extension (kg) was found to be very large for the Experimental group (d = 2.72) and large for the Control group (d = 1.20). For leg curl (kg), the effect was very large for both the Experimental group (d = 2.88) and the Control group (d = 2.25). Regarding bench press (kg), the effect was very large for the Experimental group (d = 3.51) and also very large for the Control group (d = 2.43). The intervention’s impact on assisted pull-up (kg) was very large for both the Experimental group (d = 2.77) and the Control group (d = 3.03). For the 30-s push-up test, the effect was large for the Experimental group (d = 1.27) and large for the Control group (d = 1.45). Lastly, in the 30-s sit-up test, the effect was very large for the Experimental group (d = 3.65) and large for the Control group (d = 1.46).

**TABLE 5:** Comparison of pre-test and post-test values for within-group maximum strength measurements and muscular endurance.

Parameters	Group	Pre test $\bar{X}$ ± SS	Post test $\bar{X}$ ± SS	Cohen d
Leg Extension (kg)	Experimental Group	83.93 ± 4.87	96.07 ± 4.01	2.72
	Control Group	85.71 ± 4.75	91.43 ± 4.82	1.20
Leg Curl (kg)	Experimental Group	99.29 ± 7.81	118.21 ± 5.04	2.88
	Control Group	81.43 ± 6.02	95.36 ± 6.34	2.25
Bench Press (kg)	Experimental Group	66.43 ± 6.91	87.14 ± 4.69	3.51
	Control Group	76.79 ± 3.17	83.57 ± 2.34	2.43
Assist. Pull Up (kg)	Experimental Group	53.93 ± 5.25	68.21 ± 5.04	2.77
	Control Group	53.57 ± 3.06	62.14 ± 2.57	3.03
Push Up (30 s)	Experimental Group	24.66 ± 2.53	27.04 ± 0.81	1.27
	Control Group	22.67 ± 1.16	24.13 ± 0.83	1.45
Sit Up (30 s)	Experimental Group	19.21 ± 0.97	23.36 ± 1.28	3.65
	Control Group	19.71 ± 1.68	21.86 ± 1.23	1.46

*p < 0.05; Effect Size (Cohen d): Insignificant <0.2; Small = 0.2–0.59; Moderate = 0.60–1.19; Large = 1.20–1.99; Very Large = 2.0–3.99; Close to Perfect >4.0.

Table 6 presents the findings from the repeated measures two-way ANOVA test comparing the effects of the intervention on body composition over time between the Experimental and Control groups. According to the results, statistically significant differences were observed between the groups over time in all physiological characteristics except for body weight (kg) (p < 0.05).

**TABLE 6:** Comparison of physiological characteristic differences over time between groups.

Parameters	Group	Pre test $\bar{X}$ ± SS	Post test $\bar{X}$ ± SS	Group × time interaction		
				F	p	η^2^
Body Weight (kg)	Experimental Group	75.46 ± 2.61	70.07 ± 2.63	0.181	0.674	0.007
	Control Group	75.73 ± 3.59	70.77 ± 2.81			
Body Mass İndex (kg/m2)	Experimental Group	21.94 ± 0.58	19.14 ± 0.59	**26.000**	0.001*	**0.526**
	Control Group	21.17 ± 0.64	19.84 ± 0.85			
Body Fat Percentage (%)	Experimental Group	15.36 ± 0.62	12.13 ± 0.47	**4.495**	0.044*	**0.147**
	Control Group	15.73 ± 0.51	13.20 ± 0.81			

*p < 0.05; η2: small >0.01, medium ≥0.06, large ≥0.14.

Table 7 presents the findings from the repeated measures two-way ANOVA test comparing the effects on Maximum Strength Measurements and Muscular Endurance performance parameters over time between the Experimental and Control groups. According to the results, statistically significant differences between the groups over time were observed in leg extension (kg), bench press (kg), assisted pull-up (kg), and sit-up (30 s) performance (p < 0.05).

**TABLE 7:** Comparison of differences in maximum strength measurements and muscular endurance parameters over time between groups.

Parameters	Group	Pre test $\bar{X}$ ± SS	Post test $\bar{X}$ ± SS	Group × time interaction		
				F	p	η^2^
Leg Extension (kg)	Experimental Group	83.93 ± 4.87	96.07 ± 4.01	**40.798**	0.001*	**0.611**
	Control Group	85.71 ± 4.75	91.43 ± 4.82			
Leg Curl (kg)	Experimental Group	99.29 ± 7.81	118.21 ± 5.04	3.209	0.085	0.110
	Control Group	81.43 ± 6.02	95.36 ± 6.34			
Bench Press (kg)	Experimental Group	66.43 ± 6.91	87.14 ± 4.69	**42.892**	0.001*	**0.623**
	Control Group	76.79 ± 3.17	83.57 ± 2.34			
Assist. Pull Up (kg)	Experimental Group	53.93 ± 5.25	68.21 ± 5.04	**8.320**	0.008*	**0.242**
	Control Group	53.57 ± 3.06	62.14 ± 2.57			
Push Up (30 s)	Experimental Group	24.66 ± 2.53	27.04 ± 0.81	1.825	0.188	0.066
	Control Group	22.67 ± 1.16	24.13 ± 0.83			
Sit Up (30 s)	Experimental Group	19.21 ± 0.97	23.36 ± 1.28	**11.851**	0.002*	**0.313**
	Control Group	19.71 ± 1.68	21.86 ± 1.23			

*p < 0.05; η2: small >0.01, medium ≥0.06, large ≥0.1.

## Discussion

The findings of this study indicate that a 12-week Functional Strength Training (FST) program led to significant improvements in muscle strength, muscular endurance, and body composition in elite male field hockey players. The findings demonstrated significant improvements across several parameters, particularly in the experimental group. The findings from the current study provide compelling evidence regarding the significant impact of the intervention on both physiological characteristics and muscular performance in the Experimental and Control groups. As outlined in Table 4, the intervention produced very large effects on body weight (kg) for the Experimental group (d = 2.06) and large effects for the Control group (d = 1.54). Additionally, body mass index (BMI) was significantly impacted, with near-perfect effects observed for the Experimental group (d = 4.79) and large effects for the Control group (d = 1.77). The intervention’s effect on body fat percentage was particularly striking, with near-perfect improvements for the Experimental group (d = 5.87) and very large effects for the Control group (d = 3.74). In terms of muscular strength and endurance, as presented in Table 5, both groups experienced substantial gains. The Experimental group saw very large effects in leg extension (d = 2.72), bench press (d = 3.51), and assisted pull-up (d = 2.77) performance, while the Control group showed large to very large effects across the same parameters (d = 1.20 for leg extension, d = 2.43 for bench press, and d = 3.03 for assisted pull-up). Notably, the 30-s sit-up test also revealed a very large effect for the Experimental group (d = 3.65) and large effects for the Control group (d = 1.46), further confirming the intervention’s positive impact on muscular endurance. Table 6 presents the results from the repeated measures two-way ANOVA test, which showed statistically significant differences between the groups over time in all physiological characteristics, with the exception of body weight (p < 0.05). Similarly, Table 7 shows that significant differences were observed over time in maximum strength measurements and muscular endurance performance, specifically in leg extension, bench press, assisted pull-up, and sit-up performance (p < 0.05). These findings collectively suggest that the intervention had a robust and sustained impact on both body composition and muscular performance, with particularly notable improvements in the Experimental group across all measured parameters.

These results suggest that FST can lead to positive changes, especially in strength improvements in both the lower and upper extremities, muscular endurance, and body composition in elite field hockey players. These findings are consistent with previous studies that report positive effects of functional training on overall motor skill performance (Bashir et al., 2024; Feito et al., 2018).

### Biomechanical and neuromuscular adaptations affecting muscle strength

From the perspective of exercise physiology, the strength gains reflecting adaptive responses of the neuromuscular system to applied resistance must be considered within the framework of resistance training-specific adaptation mechanisms. The effects of resistance training on strength gains are explained by increases in motor unit activation, muscle fiber hypertrophy, and neuromuscular efficiency (Aagaard et al., 2002).

It is known that in the early stages of training, strength gains are primarily associated with neural adaptations, while muscle fiber hypertrophy contributes to the process over a longer period (Enoka and Duchateau, 2015; Schoenfeld, 2010). In this process, the increase in synaptic activity at supraspinal centers, strengthening of corticospinal pathways, and improvements in motor unit synchronization are part of the neural adaptation mechanisms of resistance training (Behm et al., 2015). Furthermore, mechanical tension created by high-intensity loads not only leads to increases in maximal and explosive strength (Balshaw et al., 2022; Maden-Wilkinson et al., 2021) but also activates the mTOR pathway to stimulate muscle protein synthesis, particularly leading to hypertrophy in type II muscle fibers (Robinson et al., 2024; Haff and Triplett, 2016; Markovic and Mikulic, 2010; Zemková and Zapletalová, 2022; Gonzalo-Skok et al., 2022; Zhang et al., 2023; Carvalho et al., 2014; Hoffman, 2019; Nagahara et al., 2024; Andersen and Aagaard, 2010; Fry, 2004; Tortu et al., 2024; West et al., 2013; Montgomery, 2006; Santos et al., 2014; Schoenfeld, 2013). In this context, FST may accelerate neuromuscular adaptations by placing more mechanical load on the musculoskeletal system through dynamic, multi-joint movements, thereby contributing to increased strength production (Haff and Triplett, 2016; Markovic and Mikulic, 2010). FST has been shown to trigger a broader spectrum of neuromuscular adaptations by supporting motor unit activation and intramuscular coordination mechanisms (Haff and Triplett, 2016). The findings of the present study also indicate that FST positively impacts strength gains by improving muscle activation dynamics, which is consistent with previous studies in the literature (Xiao et al., 2025; Hovsepian et al., 2021).

The application of strength gains to functional performance is as crucial as the development of strength itself. According to the Joint-by-Joint Model, strengthening core muscles can improve force transfer between the lower and upper extremities by enhancing postural stability (Zemková and Zapletalová, 2022). This process can lead to optimal performance gains by mobilizing the produced force more efficiently. Unilateral exercises, which activate stabilizing muscles to a greater extent, may play an important role in force transfer (Gonzalo-Skok et al., 2022; Zhang et al., 2023). Although core stabilization and motor unit activation were not directly measured in this study, the significant strength increases observed in both extremities suggest that unilateral exercises may contribute to functional force transfer. Furthermore, given the literature indicating that FST supports acceleration, sprinting, and reactive force development (Carvalho et al., 2014; Hoffman, 2019), the strength increases identified in this study may support potential improvements in speed and explosive strength skills. However, since sprinting and explosive strength were not directly measured in this study, future research should include measurements such as sprint duration, reactive force (RFD), and vertical jump to clarify their effects on sprint performance and agility. This highlights the need for comprehensive studies to enhance explosive strength skills in field hockey players, who frequently reach high speeds (Andersen and Aagaard, 2010).

### Muscle fiber type transformation and endurance performance

Fast-twitch muscle fibers (Type II, particularly MyHC IIX) have a high force production capacity but are limited in endurance (Fry, 2004; Tortu et al., 2024). However, Type IIA fibers optimize both strength and endurance capacities, contributing to versatile performance (Fry, 2004; Tortu et al., 2024; West et al., 2013). High-intensity methods with short rest intervals, such as FST, can trigger the transformation of MyHC IIX to MyHC IIA, thereby enhancing muscles’ ability to generate quick force and sustain endurance (Hoffman, 2019). This adaptation can provide an advantage in sports like field hockey, which involves variable tempo. However, since muscle fiber types were not directly measured in this study, further research is needed to verify the effects of this transformation process.

Explosive force production and its sustainability are supported by the ATP-PC and anaerobic glycolysis systems (West et al., 2013). FST may stimulate metabolic power, muscle mass, and anaerobic capacity development (Montgomery, 2006).

In field hockey, considering the high-volume running demands of midfield players (Hoffman, 2019; Nagahara et al., 2024), these adaptations can enhance both explosive strength and endurance (Andersen and Aagaard, 2010). The significant increase in muscular endurance observed in the 30-s push-up and 30-s sit-up tests is consistent with findings in the literature. Although the physiological process described could explain the results of this study, it requires further investigation through aerobic endurance and sprint performance field tests for a more comprehensive evaluation.

### Body composition

Studies on body composition show that modern athletes have higher body mass indices than their predecessors, a change associated with genetic proficiency, improvements in training methodologies, nutritional approaches, and the evolution of sports dynamics (Santos et al., 2014). This change, reflecting an increase in muscle mass and a decrease in body fat percentage, is emphasized as advantageous in many sports (Schoenfeld, 2013). Research indicates that high body fat percentage negatively correlates with VO_2_max and endurance and may lead to lower scores in tests such as the 30 m sprint and vertical jump (Sharma et al., 2023). While low body fat percentage provides an advantage for overall performance, high muscle mass increases Peak Power (PP) and Mean Power (MP) values, but excessive lean mass and muscle mass may negatively affect endurance due to increased oxygen consumption (Stanforth et al., 2014). According to the body composition results of this study, FST has a substantial impact on reducing body fat percentage and improving BMI in elite field hockey players. Similar results reported in studies with field hockey players (Stewart and Sutton, 2012; Suchomel et al., 2016) show that FST-based training programs promote fat loss alongside increased lean body mass (LBM), leading to improvements in balance, coordination, speed, and agility. Our findings emphasize the positive correlation between body composition and athletic performance, which is supported by the literature. However, further laboratory-based physiological measurements are needed to confirm the reliability of these results.

## Conclusion

This study provides a significant contribution to the literature, as it is one of the few that investigates the effects of FST on the physical performance of elite field hockey players. The results demonstrate the positive effects of FST on muscle strength, muscular endurance, and body composition in these athletes. The findings suggest that FST could be an effective method for enhancing physical performance in field hockey. However, additional field-based research including specific tests on speed, acceleration, and agility is needed to determine its effect on these performance metrics. Coaches and sports scientists may consider FST as a means to regulate metabolic processes through aerobic and anaerobic loading techniques, thus contributing to fundamental motor skills and aligning these gains with sport-specific demands.

## Practical applications

FST requires movement control and stabilization in both unison and bilateral movements. Therefore, it is highly recommended for the development of strength in the lower and upper extremities. Based on the findings of this study, FST is a practical approach for improving fitness, strength, endurance, and body composition. It can be incorporated into field hockey training programs to optimize the effects of strength development and improve overall physical performance, particularly in strength training exercises. Moreover, given its adaptability to different fitness levels, FST can be customized to meet individual training requirements and be used in daily training for elite athletes. Future research should examine FST’s effects on other performance metrics such as sprint time, vertical jump, and agility, and its implementation in long-term training plans.

## Data Availability

The raw data supporting the conclusions of this article will be made available by the authors, without undue reservation.
